# Supplementary material for: The Venice specimen of Ouranosaurus nigeriensis (Dinosauria, Ornithopoda)
Source: PeerJ. 2017 Jun 20;5:e3403. doi: 10.7717/peerj.3403 (PMC5480399; doi:10.7717/peerj.3403)
Supplement: Supplemental Information 1 [file peerj-05-3403-s001.docx]

**THE VENICE SPECIMEN OF *OURANOSAURUS NIGERIENSIS* (DINOSAURIA, ORNITHOPODA)**

Filippo Bertozzo^1^, Fabio Marco Dalla Vecchia^2,4^, Matteo Fabbri^3^

1 Earth System Science-AMGC, Vrije Universiteit Brussel, Pleinlaan 2, Brussels/Bruxelles, Belgium

2 Institut Català de Paleontologia Miquel Crusafont, Carrer de l’Escola Industrial 23, E-08201 Sabadell, Spain.

3 Department of Geology and Geophysics, Yale University, 210 Whitney Ave., New Haven, CT06611, USA
4 Current affiliation: Soprintendenza Archeologia, Belle Arti e Paesaggio del Friuli Venezia Giulia, Nucleo Operativo di Udine, Via Zanon 22, I-33100 Udine, Italy

Corresponding Author: Filippo Bertozzo

Email: filippo.bertozzo@gmail.com

**SUPPORTING INFORMATION**

Figure 1. SI1. Measurement locations reported in Table 1 SI taken on vertebrae (A), coracoid (B), scapula (C), sternal plate (D), humerus (E), ulna (F), radius (G) , metacarpals and manus phalanges (H), ilium (I), ischium (J), femur (K), fibula (L), tibia (M), and metatarsals and pedal phalanges (N). Abbreviations: H, height; Hns, height of the neural spine; L, length; W, width. Elements are not drawn to scale.


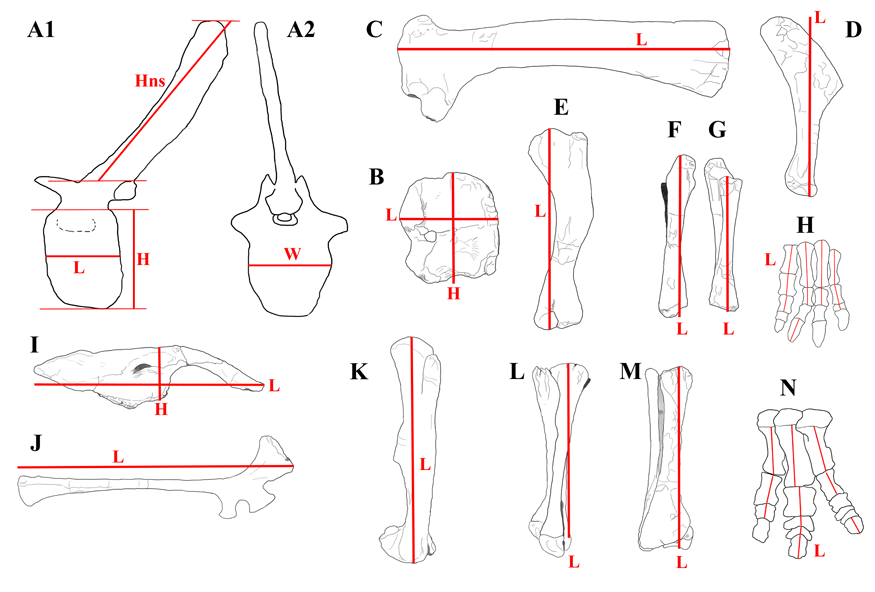


Table 1. SI2. Measurements of the skeletal elements of MSNV3714, *Ouranosaurus nigeriensis*. Legend: *, measurements that are unreliable because of reconstruction; - , missing element; As, astragalus; c., vertebral centrum; ca, calcaneum; CdV, caudal vertebra; Co, coracoid; CV, cervical vertebra; DV, dorsal vertebra; Fe, femur; Fi, fibula; H, height; Hm, hemapophysis; Hu, humerus; Il, ilium; int, intermedium; Is, ischium; L, length; McII-V, metacarpals II-V; MtII-IV, metatarsals II-IV; ns., neural spine; P. GIRDLE, pelvic girdle; Ph, manus phalanges; Pph, pedal phalanges; Pu, pubis; rad, radiale; Sc, scapula; S. GIRDLE, shoulder girdle; St, sternal plate; Ti, tibia; Ul, ulnare; V, sacral vertebra; W, width. Left elements are reported within brackets. Measurements are in millimetres. Measurement locations given in figure 1 SI. Heights of neural spines are those of the restored spines (i.e., including artificial parts).

| **AXIAL** |  |  |  |  |  |
| --- | --- | --- | --- | --- | --- |
| Axis c. L | * | Axis c.W | 55 | Axis c. H | 140 |
| **CV3** c.L | 87 | CV3 c.W | 65 | CV3 c.H | 54 |
| CV4 c.L | 88 | CV4 c.W | 60 | CV4 c.H | 75 |
| CV5 c.L | 92 | CV5 c.W | 70 | CV5 c.H | 74 |
| CV6 c.L | 91 | CV6 c.W | 80 | CV6 c.H | 80 |
| CV7 c.L | 100 | CV7 c.W | 80 | CV7 c.H | 80 |
| CV8 c.L | 110 | CV8 c.W | 85 | CV8 c.H | 85 |
| CV9 c.L | 110 | CV9 c.W | 85 | CV9 c.H | 83 |
| CV10 c.L | 100 | CV10 c.W | 85 | CV10 c.H | 85 |
| CV11 c.L | 100 | CV11 c.W | 80 | CV11 c.H | 85 |
| **DV1** c.L | 97 | DV1 c.W | 80 | DV1 c.H | 80 |
| DV1 ns.H | 113 | DV2 c.L | 100 | DV2 c.W | 74 |
| DV2 c.H | 76 | DV2 ns.H | 300 | DV3 c.L | 98 |
| DV3 c.W | 70 | DV3 c.H | 78 | DV3 ns.H | 450 |
| DV4 c.L | 98 | DV4 c.W | 70 | DV4 c.H | 80 |
| DV4 ns.H | 600 | DV5 c.L | 98 | DV5 c.W | 70 |
| DV5 c.H | 88 | DV5 ns.H | 610 | DV6 c.L | 100 |
| DV6 c.W | 75 | DV6 c.H | 85 | DV6 ns.L | 645 |
| DV7 c.L | 92 | DV7 c.W | 82 | DV7 c.H | 90 |
| DV7 ns.H | 635 | DV8 c.L | 100 | DV8 c.W | 75 |
| DV8 c.H | 88 | DV8 ns.H | 650 | DV9 c.L | 97 |
| DV9 c.W | 75 | DV9 c.H | 90 | DV9 ns.H | 660 |
| DV10 c.L | * | DV10 c.W | * | DV10 c.H | * |
| DV10 ns.H | 640 | DV11 c.L | * | DV11 c.W | * |
| DV11 c.H | * | DV11 ns.H | 610 | DV12 c.L | 87 |
| DV12 c.W | 100 | DV12 c.H | 110 | DV12 ns.H | 580 |
| DV13 c.L | 100 | DV13 c.W | 90 | DV13 c.H | 108 |
| DV13 ns.H | 580 | DV14 c.L | 106 | DV14 c.W | 90 |
| DV14 c.H | 100 | DV14 ns.H | 580 | DV15 c.L | * |
| DV15 c.W | * | DV15 c.H | * | DV15 ns.H | 540 |
| DV16 c.L | 105 | DV16 c.W | 86 | DV16 c.H | 97 |
| DV16 ns.H | 480 | DV17 c.L | 112 | DV17 c.W | 92 |
| DV17 c.H | 85 | DV17 ns.H | 450 | **SV1** c.L | 105 |
| SV1 c.W | 100 | SV1 c.H | 120 | SV1 ns.H | 490 |
| SV2 c.L | 126 | SV2 c.W | * | SV2 c.H | * |
| SV2 ns.H | 490 | SV3 c.L | 120 | SV3 c.W | * |
| SV3 c.H | * | SV3 ns.H | 490 | SV4 c.L | 113 |
| SV4 c.W | * | SV4 c.H | * | SV4 ns.H | 495 |
| SV5 c.L | 127 | SV5 c.W | * | SV5 c.H | * |
| SV5 ns.H | 510 | SV6 c.L | 100 | SV6 c.W | 140 |
| SV5 c.H | 130 | SV6 ns.H | 500 | **CdV**1 c.L | 86 |
| CdV1 c.W | 120 | CdV1 c.H | 115 | CdV1 ns.H | 465 |
| CdV2 c.L | 82 | CdV2 c.W | 115 | CdV2 c.H | 100 |
| CdV2 ns.H | 460 | CdV3 c.L | 79 | CdV3 c.W | 120 |
| CdV3 c.H | 120 | CdV3 ns.H | 430 | CdV4 c.L | 82 |
| CdV4 c.W | 110 | CdV4 c.H | 140 | CdV4 ns.H | 370 |
| CdV5 c.L | 82 | CdV5 c.W | 105 | CdV5 c.H | 130 |
| CdV5 ns.H | 350 | CdV6 c.L | 79 | CdV6 c.W | 100 |
| CdV6 c.H | 134 | CdV6 ns.H | 310 | CdV7 c.L | 75 |
| CdV7 c.W | 95 | CdV7 c.H | 120 | CdV7 ns.H | 305 |
| CdV8 c.L | 75 | CdV8 c.W | 90 | CdV8 c.H | 120 |
| CdV8 ns.H | 265 | CdV9 c.L | 75 | CdV9 c.W | 90 |
| CdV9 c.H | 115 | CdV9 ns.H | 270 | CdV10 c.L | 76 |
| CdV10 c.W | 90 | CdV10 c.H | 110 | CdV10 ns.H | 200 |
| CdV11 c.L | 76 | CdV11 c.W | 86 | CdV11 c.H | 105 |
| CdV11 ns.H | 226 | CdV12 c.L | 75 | CdV12 c.W | 85 |
| CdV12 c.H | 100 | CdV12 ns.H | 190 | CdV13 c.L | 75 |
| CdV13 c.W | 80 | CdV13 c.H | 90 | CdV13 ns.H | 170 |
| CdV14 c.L | 75 | CdV14 c.W | 85 | CdV14 c.H | 92 |
| CdV14 ns.H | 131 | CdV15 c.L | 76 | CdV15 c.W | 85 |
| CdV15 c.H | 87 | CdV15 ns.H | 157 | CdV16 c.L | 75 |
| CdV16 c.W | 88 | CdV16 c.H | 82 | CdV16 ns.H | 141 |
| CdV17 c.L | 77 | CdV17 c.W | 85 | CdV17 c.H | 80 |
| CdV17 ns.H | 140 | CdV18 c.L | 78 | CdV18 c.W | 82 |
| CdV18 c.H | 75 | CdV18 ns.H | 137 | CdV19 c.L | 77 |
| CdV19 c.W | 78 | CdV19 c.H | 75 | CdV19 ns.H | 132 |
| CdV20 c.L | 77 | CdV20 c.W | 77 | CdV20 c.H | 75 |
| CdV20 ns.H | 132 | CdV21 c.L | 76 | CdV21 c.W | 75 |
| CdV21 c.H | 75 | CdV21 ns.H | 130 | CdV22 c.L | 77 |
| CdV22 c.W | 65 | CdV22 c.H | 71 | CdV22 ns.H | 115 |
| CdV23 c.L | 77 | CdV23 c.W | 68 | CdV23 c.H | 70 |
| CdV23 ns.H | 114 | CdV24 c.L | 76 | CdV24 c.W | 67 |
| CdV24 c.H | 69 | CdV24 ns.H | 77 | CdV25 c.L | 75 |
| CdV25 c.W | 62 | CdV25 c.H | 68 | CdV25 ns.H | * |
| CdV26 c.L | 75 | CdV26 c.W | 62 | CdV26 c.H | 68 |
| CdV26 ns.H | * | CdV32 c.L | 59 | CdV32 c.W | 38 |
| CdV32 c.H | 40 | CdV32 ns.H | * | CdV33 c.L | * |
| CdV33 c.W | * | CdV33 c.H | * | CdV33 ns.H | * |
| CdV34 c.L | 58 | CdV34 c.W | 35 | CdV34 c.H | 35 |
| CdV34 ns.H | * | CdV35 c.L | 50 | CdV35 c.W | 31 |
| CdV35 c.H | 31 | CdV35 ns.H | * | CdV36 c.L | 50 |
| CdV36 c.W | 25 | CdV36 c.H | 28 | CdV36 ns.H | * |
| CdV37 c.L | 43 | CdV37 c.W | 20 | CdV37 c.H | 26 |
| CdV37 ns.H | * | CdV38 c.L | 47 | CdV38 c.W | 20 |
| CdV38 c.H | 25 | CdV38 ns.H | * | **Hm**1 L | 365 |
| Hm2 L | 360 | Hm3 L | 330 | Hm4 L | 320 |
| Hm5 L | 290 | Hm6 L | * | Hm7 L | 270 |
| Hm8 L | 255 | Hm9 L | 230 | Hm10 L | 220 |
| Hm11 L | 200 | Hm12 L | 200 | Hm13 L | 200 |
| Hm14 L | 155 | Hm15 L | 175 | Hm16 L | 165 |
| Hm17 L | 130 | Hm18 L | 110 | Hm19 L | - |
| Hm20 L | 95 | Hm21 L | - | Hm22 L | - |
| Hm23 L | 80 | Hm24 L | - | Hm25 L | - |
| **S. GIRDLE** |  |  |  |  |  |
| (Co) L | 125 | (Co) H | 130 | Sc L | 630 |
| (Sc) L | 640 | St L | 330 | (St) L | 330 |
| **FORELIMB** |  |  |  |  |  |
| Hu L | 510 | (Hu) L | 510 | U L | 410 |
| (U) L | 410 | Ra L | 350 | (Ra) L | 340 |
| (Rad+int) L | 40+22 | (Ul) L | 46 | McII L | 85 |
| McIII L | 113 | McIV L | 108 | McV L | 68 |
| PhII-1 L | 46 | PhII-2 L | 15 | (PhII-3) L | 55 |
| PhIII-1 L | 40 | (PhIII-3) L | 63 | PhIV-1 L | 45 |
| (PhIV-2) L | 50 | PhIV-3 L | 57 | (PhIV-3) L | 53 |
| PhV-1 L | 49 | (PhV-1) L | 51 | PhV-2 L | 42 |
| **P. GIRDLE** |  |  |  |  |  |
| Il L | * | Il H | 176 | (Il) L | 770 |
| (Il) H | 176 | Pu L | * | (Pu) L | * |
| Is L | 880 | (Is) L | 880 |  |  |
| **HIND LIMB** |  |  |  |  |  |
| Fe L | 920 | Ti L | 710 | (Ti) L | 710 |
| Fi L | 690 | (Fi) L | 650 | (Mt II) L | 185 |
| (Mt III) L | 215 | (Mt IV) L | 190 | (PphII-1) L | 100 |
| (PphII-2) L | 60 | (PphIII-1) L | 95 | (PphIII-2) L | 42 |
| (PphIII-3) L | 28 | (PphIII-4) L | 70 | (PphIV-1) L | 85 |
| (PphIV-5) L | 47 |  |  |  |  |

**Notes on the two field maps figured in Text Fig. 2. SI3**

Sheet 1 stored at the MNHN contains the field map of the paratype skeleton from the tip of the tail to the first dorsal vertebra on the front side, while it contains the field map of the neck of the paratype with part of the appendicular skeleton on the back side (R Allain, pers. comm., 2017). It includes the bones numbered from 1 to 137. The elements with the numbers 96 (a bone near the ulna) and 97 (a fragment of ilium) are not reported in the map, but they are mentioned in a handwritten note (see Text-Fig. 2). A distal caudal vertebra also lacks a number and was not drawn in the map, but it is mentioned in a handwritten note (see Text-Fig. 2).

Sheet 2 contains the field map of some elements from the pelvic region, part of the tail, some limb bones and likely some dorsal vertebrae on the front side, while a few other elements are mapped on the rear side of the sheet (R Allain, pers. comm., 2017). The bones on the front side of the map are numbered 200, 202-209, 212, 214-231, 233-256 and 258-286), whereas those on the back side bear the numbers 210-211, 213 and 257. Some numbers (e.g., 201, 213 and 232) are apparently missing in the sheet, assuming a progressive numeration of the bones.

There is no indication of the relative location to each other sheet. P. Taquet is no longer sure about their relative placement (R Allain, pers. comm., 2016).

Specimens corresponding to numbers 138-199 do not occur in the two sheets, suggesting that part of the collected bones were not mapped in the two sheets. Possibly, the two sheets represent the field maps of different spots with fossil remains in the same locality, while a further sheet containing specimens 138 to 199 is missing.

In Text-Fig. 2, the original field identification of the bones reported in the field maps are translated into English and sometimes they are abbreviated. The original identification of the skeletal element is written in dark gray instead of black colour when it is different from our identification or we doubt its correctness. Some of the handwritten notes are also reported translated into English and in dark gray colour. The sheets do not contain scale bars and assembled parts are not exactly at the same scale. Lengths of some skeletal elements are reported.

**Phylogenetic analysis. SI4**

Modified characters of the matrix from McDonald (2012), emended according to McDonald et al. (2012b).

**Original ch. 98** (97 in TNT). Sternal, caudomedial process: absent (0); present (1).

McDonald (2012) coded this character as missing (?). The process is not preserved in the holotype because the sternals are damaged. The sternals of the Venice specimen preserve that process, thus the coding is 1.

**Original ch. 99** (98) - Sternal, shape of main body in dorsal or ventral view, excluding caudolateral process if present: convex medially and concave laterally (0); convex medially and straight laterally (1).

This character was deleted after suggestion by A. McDonald (A McDonald, pers. comm., 2017).

**Original ch. 101** (100). Scapula, expansion of caudal end: gently convex expansion along caudodorsal margin, caudoventral margin tapers into hook-like flange (0); caudal end paddle-shaped, dorsal and ventral margins of scapula diverge towards caudal end (1); caudal margin of scapula straight, dorsal and ventral margins are parallel approaching caudal margin of scapula and meet caudal margin at nearly right angles (2).

McDonald (2012) coded this character 2. However, the dorsal and ventral margins of the scapulae diverge towards caudal end in both specimens. Furthermore, the distal part of the blade is preserved in the left scapula of MSNVE 3714 (see Fig. 13E), thus, the coding is 1.

**Original ch. 102** (101). - Scapula, shape of acromion process in lateral view: subtriangular (0); cranial margin of process is convex (1); low, rounded protuberance (2).

This character was deleted after suggestion by A. McDonald (A McDonald, pers. comm., 2017).

**Original ch. 107** (106). Manus, arrangement of metacarpals II-IV: spreading (0); closely appressed (1) (You et al*.*, 2003b, character 49).

McDonald (2012) coded this character 1. Metacarpals (see Fig. 15B) do not show the lateral and medial facets for their reciprocal articulation as the metacarpals II-IV of *Iguanodon bernissartensis* (see text), therefore they were not closely appressed liked those of *I. bernissartensis* but somewhat spreading. Thus, the coding of this character is 0.

**Original ch. 112** (111). Ilium, dorsal margin above pubic and ischial peduncles and acetabulum: straight (0); convex (1); sinuous, convex above pubic peduncle and concave above ischial peduncle (2) (modified from Weishampel et al*.*, 2003, character 55).

McDonald (2012) coded this character 1 because the margin is convex in the holotype. However, the dorsal margin is straight in the Venice specimen (see Fig. 16A-16B). Thus, its coding is 0/1.

**Original ch. 120** (119). Ischium, morphology of shaft: curved caudally (0); curved cranially (1); straight (2) (modified from Norman, 2002, character 60).

McDonald (2012) coded this character 1. The shaft is curved cranially in the holotype, whereas it is straight in the Venice specimen, thus, the states of the character are 1/2.

**Original ch. 123** (122). Femur, groove on caudal aspect of femoral head: present (0); absent (1) (Winkler et al., 1997, character 25).

McDonald (2012) coded this character as missing. The state is considered here to be 1 because there is no groove in the Venice specimen.

**Original ch. 131** (130). Pes, morphology of unguals on digits II-IV: dorsoventrally flattened, but elongate and pointed (0); dorsoventrally flattened and elongate, but with blunt truncated tips (1); hoof-like shape (2) (modified from Norman, 2002, character 67).

McDonald (2012) coded this character 1. The ungual of digit III is midway between state 1 and 2. The character state 2 is better described in Norman (2015, character 105, state 2). It is coded 2 here according to the better fit of ungual of digit III to the definition of state 2 in Norman (2015).

**Original ch.** 133 (132). Ilium, brevis fossa, transverse width: narrow (0); very broad and expanding in width towards its caudal margin such that it appears triangular in dorsal or ventral view (1) (Barrett et al., 2011, character 132).

McDonald (2012) coded this character 0. The actual condition in the Venice specimen is different from both states, thus, it is considered 'inapplicable' (-).

Modified characters of the matrix from Norman (2015).

**Original ch. 71**. Dorsal vertebrae, centrum articular surfaces: anterior dorsals amphiplatyan (0), anterior dorsals ‘cervicalized’ and display moderate opisthocoely, before becoming more regularly amphiplatyan about one-third of the way along the dorsal series (1), entire dorsal series displays moderate opisthocoely (2).

**Modified ch. 71**. Dorsal vertebrae, centrum articular surfaces: anterior dorsals amphiplatyan (0), anterior dorsals ‘cervicalized’ and display moderate opisthocoely, before becoming more regularly amphiplatyan along the dorsal series (1), entire dorsal series displays moderate opisthocoely (2).

**Explanation**: Only the first two dorsals are opisthocoelous in MSNVE 3714, while the others are slightly amphicoelous to amphiplatyan. Therefore, "about one-third of the way" was eliminated from the definition of state 1.

The coding 1 for *O. nigeriensis* is confirmed.

**Ch 73.** Epaxial ossified tendons: arranged in linear bundles (0), form a layered lattice against the neural spines (1) – (emended from Weishampel et al., 2003: 42).

Norman (2015) coded this character 1. As noted above, there is no available information about the epaxial ossified tendons in *O. nigeriensis*, thus, the state was considered unknown and coded as missing.

**Original ch. 80**. Ungual phalanx of manus digit I (morphology): narrow and claw-like (0), conical spike (1), enlarged and laterally compressed spine (2), small, narrow spine (3), absent (4).

**Modified ch. 80**. Ungual phalanx of manus digit I (morphology): narrow and claw-like (0), conical spike (1), enlarged and laterally compressed spine (2), small, narrow spine (3).

**Explanation**: "Absent" is not a morphology, so state 4 was cancelled and the character "Ungual phalanx of manus digit I: (0) present, (1) absent" replaces character 85 (see below). Character 80 is coded 2, although the ungual phalanx of manus digit I is not compressed laterally but has just a flat ?palmar face.

**Ch. 81**. Manus phalanx 1 of digit I (morphology): normal phalangeal proportions (0), discoidal plate (1), absent (2).

Norman (2015) coded this character 1. The state is considered here to be unknown because phalanx I-1 of the manus is not preserved in *O. nigeriensis*. Consequently, the state was coded as missing.

**Ch. 83**. Metacarpals II−IV: capable of forming a broad ‘spreading’ palm (0), robust, compressed against adjacent metacarpals (1), slender and elongate (2). (Norman, 2002: 50).

Norman (2015) coded this character 1. Metacarpals II-IV of *O. nigeriensis* were not "compressed against adjacent metacarpals" as those of *I. bernissartensis* and were instead somewhat spreading (see text)*.* They are slenderer than the corresponding metacarpals of *I. bernissartensis* and less elongated than those of *M. atherfieldensis*. Consequently, the state of this character is coded 0.

**Original ch. 85**. Ungual of manus digit I: claw-like (0), subconical (1), absent (2). (Norman, 2002: 52).

**Modified ch. 85**. Ungual phalanx of manus digit I: (0) present, (1) absent.

**Explanation:** This character is redundant with character 80. According to the changes done in character 80, we replace character 85 with a new character accounting for the presence or absence of the ungual phalanx of manus digit I. *O. nigeriensis* is coded 0.

**Ch. 87**. Manus digit III: four phalanges (0), three phalanges (1). (Sereno, 1986).

Norman (2015) coded this character 1. The state is considered here as unknown because the phalanges of *O. nigeriensis* that are referred to manus digit III were scattered in the field and their number in the mount is interpretative. Consequently, *O. nigeriensis* is coded as missing

**Ch. 90.** Ilium, dorsal margin development: no transverse thickening of the dorsal edge in the region above the ischial peduncle (0), transversely thickened, bevelled edge (1), thickened dorsal edge developed into a rolled edge (2), discrete bulbous boss present posterodorsal to the ischiadic peduncle (3), prominently everted and downturned (flap-shaped) pendule that overhangs the ischiadic peduncle region of the ilium (4) – (emended from Norman, 2002: 56).

Norman (2015) coded this character 1. The dorsal margin of the ilia of *O. nigeriensis* specimens bears the lateral structure that is described as "knob-like supracetabular process (antitrochanter)" in the text of this paper (see also Fig. 16B and 16D-16E). The dorsal margin of the ilium of *M. atherfieldensis* (coded 1 by Norman, 2015) does not preserve the same structure (see Norman 1986, figs. 53-54). However, the knob of *O. nigeriensis* appears to be less prominent than that present in *Bactrosaurus johnsoni* (coded 3 by Norman, 2015). Here, the state of character 90 for *O. nigeriensis* is coded 3. However, we did a separate run of the matrix with the state coded 1. The position of *O. nigeriensis* in the resulting strict consensus tree does not change.

**Original ch. 92**. Ilium, brevis fossa: arched recess on the ventral surface of the postacetabular process of the ilium enclosed laterally by a ridge (0), shallow brevis fossa no lateral ridge (1), postacetabular blade narrow and no brevis fossa present (2).

**Modified ch.** **92**. Ilium, brevis fossa: arched recess on the ventral surface of the postacetabular process of the ilium enclosed laterally by a ridge (0), shallow brevis fossa no lateral ridge (1), no brevis fossa present (2).

**Explanation:** "postacetabular blade narrow" refers to the morphology of the postacetabular blade not to that of the brevis fossa. Furthermore, the ilium of *O. nigeriensis* presents no brevis fossa, but the distal part of the postacetabular process cannot be considered either "narrow" nor a blade. Thus, "postacetabular blade narrow" was eliminated from the character state description. Norman (2015) coded this character 1. The state is coded 2 here because there is no fossa in the brevis shelf of the ilea of MSNVE3714.

**Original ch. 94**. Pubis, pubic shaft: terminates bluntly adjacent to distal end of ischium (0), slender, shorter than ischial shaft, and tapers to a point (1). (Norman, 2002: 59).

**Modified ch. 94.** Pubis, pubic shaft: terminates bluntly adjacent to distal end of ischium (0), slender, shorter than ischial shaft, and usually tapering to a point (1).

**Explanation:** Norman (2015) coded it 1 for *O. nigeriensis.* We agree, but the pubic shaft of the holotype does not taper to a point, as noticed also by McDonald (2012). Thus, the definition of the character state 1 has been modified.

**Ch. 95**. Ischium, shaft morphology: straight (0), bowed (1). (Norman, 2002: 60).

Norman (2015) coded this character 1 based on the holotype. However, the state is 0 in the Venice specimen (see Fig. 16I-16J), thus, the coding is 0/1.

**Ch. 99**. Femoral head, articular surface bears a prominent groove posteriorly: present (0), absent (1).

Norman (2015) coded this character as missing. The state is considered here to be 1 because there is no groove in the Venice specimen.

**Ch.100**. Femur, curvature of shaft: distal half of shaft curved caudally (0), straight (1). (Norman, 2002: 62).

Norman (2015) coded this character 1. We also code it 1, although the femur shows a faint distal curvature in O*. nigeriensis* (see Fig. 17A and 17C). Likely, this character is intraspecifically variable (Verdú et al., 2017).

**Ch.105**. Pedal ungual phalanges, shape: dorsoventrally flattened, but elongate and pointed (0), elongate, bluntly truncated tip with prominent claw grooves retained (1), anterior margin broadly rounded in dorsal view, lateral claw grooves either indistinct or entirely absent (2). (Norman, 2002: 67).

Norman (2015) coded this character as missing. The character is coded here 2 because the ungual phalanx of digit III does not seem to correspond to state 1 (see Fig. 18). Actually, the morphology of that ungual phalanx is midway between states 1 and 2.

**Additional references**

**Barrett PM, Butler RJ, Twitchett RJ, Hutt S. 2011**. New material of *Valdosaurus canaliculatus* (Ornithischia: Ornithopoda) from the Lower Cretaceous of southern England. *Special Papers in Palaeontology* **86**:131-163 DOI 10.1111/j.1475-4983.2011.01076.x.
